# Association between early tracheostomy and patient outcomes in critically ill patients on mechanical ventilation: a multicenter cohort study

**DOI:** 10.1186/s40560-022-00610-x

**Published:** 2022-04-11

**Authors:** Aiko Tanaka, Akinori Uchiyama, Tetsuhisa Kitamura, Ryota Sakaguchi, Sho Komukai, Tasuku Matsuyama, Takeshi Yoshida, Natsuko Tokuhira, Naoya Iguchi, Yuji Fujino

**Affiliations:** 1 Department of Anesthesiology and Intensive Care Medicine, Osaka University Graduate School of Medicine, 2-15 Yamadaoka, Suita, Osaka 565-0871 Japan; 2 Division of Environmental Medicine and Population Sciences, Department of Social and Environmental Medicine, Osaka University Graduate School of Medicine, Suita, Osaka Japan; 3 Division of Biomedical Statistics, Department of Integrated Medicine, Department of Social and Environmental Medicine, Osaka University Graduate School of Medicine, Suita, Osaka Japan; 4 Department of Emergency Medicine, Kyoto Prefectural University of Medicine, Kyoto, Japan

**Keywords:** Intensive care, Prognosis, Prolonged mechanical ventilation, Tracheostomy

## Abstract

**Background:**

Tracheostomy is commonly performed in critically ill patients because of its clinical advantages over prolonged translaryngeal endotracheal intubation. Early tracheostomy has been demonstrated to reduce the duration of mechanical ventilation and length of stay. However, its association with mortality remains ambiguous. This study aimed to evaluate the association between the timing of tracheostomy and mortality in patients receiving mechanical ventilation.

**Methods:**

We performed a retrospective cohort analysis of adult patients who underwent tracheostomy during their intensive care unit (ICU) admission between April 2015 and March 2019. Patients who underwent tracheostomy before or after 29 days of ICU admission were excluded. Data were collected from the nationwide Japanese Intensive Care Patient Database. The primary outcome was hospital mortality. The timing of tracheostomy was stratified by quartile, and the association between patient outcomes was evaluated using regression analysis.

**Results:**

Among the 85558 patients admitted to 46 ICUs during the study period, 1538 patients were included in the analysis. The quartiles for tracheostomy were as follows: quartile 1, ≤ 6 days; quartile 2, 7–10 days; quartile 3, 11–14 days; and quartile 4, > 14 days. Hospital mortality was significantly higher in quartile 2 (adjusted odds ratio [aOR]: 1.52, 95% confidence interval [CI]: 1.08–2.13), quartile 3 (aOR: 1.82, 95% CI: 1.28–2.59), and quartile 4 (aOR: 2.26, 95% CI: 1.61–3.16) (*p* for trend < 0.001) than in quartile 1. A similar trend was observed in the subgroup analyses of patients with impaired consciousness (Glasgow Coma Scale score < 8) and respiratory failure (PaO_2_:FiO_2_ ≤ 300) at ICU admission (*p* for trend = 0.081 and 0.001, respectively).

**Conclusions:**

This multi-institutional observational study demonstrated that the timing of tracheostomy was significantly and independently associated with hospital mortality in a stepwise manner. Thus, early tracheostomy may be beneficial for patient outcomes, including mortality, and warrants further investigation.

**Supplementary Information:**

The online version contains supplementary material available at 10.1186/s40560-022-00610-x.

## Background

Prolonged ventilation in critically ill patients is associated with long intensive care unit (ICU) and hospital stays and high mortality rates [1–3]. Compared to translaryngeal endotracheal intubation, tracheostomy has more potential advantages for patients with mechanical ventilation, such as lower airflow resistance and breathing effort, less administration of sedatives, better patient comfort and mobilization, weaning with less invasive attachment to and disconnection from the ventilator, and lower incidence of ventilator-associated pneumonia (VAP) [4–8]. Therefore, tracheostomy is commonly recommended for patients who are expected to require prolonged mechanical ventilation by international consensus [9, 10]. Over 20 years, the number of patients with mechanical ventilation in the ICU who underwent this procedure increased from 7 to 26% [11–13]. However, tracheostomy procedures pose a risk of complications, such as bleeding, stomal infection, and tube displacement, but these are mostly non-fatal [14, 15].

Numerous studies have been conducted concerning the optimal timing and benefits of early implementation to determine the appropriate indication for tracheostomy in critically ill patients with mechanical ventilation. In recent systematic reviews of randomized controlled trials [16–18], early tracheostomy demonstrated favorable outcomes, achieving a lower duration of mechanical ventilation and ICU stay than those of late tracheostomy. However, no association with mortality was found in these meta-analyses. Since the definition for the timing of tracheostomy varies among studies (early, within 2–10 days; late, 6–29 days or more) [19–23], direct two-arm comparisons remain challenging. Hence, evidence on the association between the timing of tracheostomy and mortality is still lacking.

Accordingly, we aimed to conduct a detailed statistical analysis of the impact of the timing of tracheostomy on the clinical outcomes of patients who received mechanical ventilation in a multicenter ICU setting.

## Methods

### Design and setting

This retrospective cohort study analyzed data from the Japanese Intensive Care PAtient Database (JIPAD), a multicenter observational data registry of critically ill patients established by the Japanese Society of Intensive Care Medicine. As of December 2019, the JIPAD study involves 70 ICUs and more than 100,000 patients in Japan. The Japanese Society of Intensive Care Medicine has a partnership agreement with the Australian and New Zealand Intensive Care Society Center for Outcome and Resource Evaluation. Consequently, the JIPAD collects data similar to those of the Australian and New Zealand Intensive Care Society Adult Patient Database (ANZICS APD), which is widely recognized as one of the largest ICU databases. A complete description of the JIPAD methodology has been previously provided [24].

### Study population

We included adult patients aged ≥ 18 years who were admitted to the ICU between April 2015 and March 2019 and underwent a tracheostomy during their ICU admission. Patients with repeat ICU admissions from the same hospital episode, patients who underwent tracheostomy before the present ICU admission or after 29 days of ICU admission, and patients with missing tracheostomy dates were excluded.

### Data collection

With the aim to improve the quality of care and develop intensive care practices, the JIPAD collects clinical data, including patient demographics, diagnosis at ICU admission, physiological data and treatment in the ICU, and patient outcomes. In this study, the following data were collected from the JIPAD: age, sex, body mass index, comorbidities (chronic heart failure, chronic respiratory failure, liver cirrhosis, liver failure, acute leukemia/multiple myeloma, lymphoma, metastatic cancer, immunosuppression, acquired immunodeficiency syndrome, and maintenance dialysis: [yes, no]), emergency admission (yes, no), surgical type of admission (yes, no), systematic diagnosis for ICU admission (cardiac, respiratory, gastrointestinal, neurological, sepsis, trauma, and other diagnoses), Acute Physiology And Chronic Health Evaluation (APACHE) II score, data within 24 h after ICU admission (incidence of acute kidney injury [yes, no], lowest PaO_2_:FiO_2_, Glasgow Coma Scale [GCS] score), length of hospital stay before ICU admission, ICU treatment (extracorporeal membrane oxygenation [yes, no], continuous renal replacement therapy [yes, no], duration of mechanical ventilation before tracheostomy, duration of mechanical ventilation in ICU, liberation of mechanical ventilation during ICU stay [yes, no]), length of ICU and hospital stay, and ICU and hospital mortality (yes, no). In the present study, immunosuppression and acquired immunodeficiency syndrome were collectively categorized under immunodeficiency. Comorbid acute leukemia/multiple myeloma, lymphoma, and metastatic cancer were categorized as malignancy. Chronic liver disease was defined as liver cirrhosis or liver failure. The data from the JIPAD used in this study, as well as from the ANZICS APD, were obtained exclusively during hospitalization at the registered institutions.

### Outcomes

The primary outcome of this study was hospital mortality, and the secondary outcome was ICU mortality.

### Statistical analyses

Continuous data were summarized as medians and interquartile ranges (IQRs), while categorical data were presented as numbers and percentages. The timing of tracheostomy starting from ICU admission was divided into quartiles: Q1, ≤ 6 days; Q2, 7–10 days; Q3, 11–14 days; and Q4, > 14 days. Differences in proportions were evaluated using the Chi-square test or Fisher’s exact test. Differences in distributed data were evaluated using the Kruskal–Wallis tests for the four groups.

To determine the relationships between the quartiles of the timing of tracheostomy and the primary and secondary outcomes, univariable and multivariable logistic regression models were developed, and the crude and adjusted odds ratios (ORs) and their 95% confidence intervals (CIs) were calculated. Age (1-year increment), sex (male, female), and APACHE II score (1-point increment) were added to the multivariable model for hospital and ICU mortality to adjust for potential confounders. We described the possible non-linear associations between the timing of tracheostomy and the estimated hospital and ICU mortality using restricted cubic splines in the univariable logistic regression model. Moreover, we performed a planned subgroup analysis stratified by the level of consciousness at ICU admission (GCS score < 8 or ≥ 8). Considering the effect of the timing of tracheostomy on lung injury, a subgroup analysis was performed in patients with respiratory failure. Respiratory failure was defined as the lowest PaO_2_:FiO_2_ ≤ 300 during the first 24 h of ICU admission; patients admitted to the ICU for cardiac diagnosis were excluded from the subgroup with respiratory failure. We also conducted a subgroup analysis of patients admitted to the ICU after cardiovascular surgery. The interaction effect between the timing of tracheostomy and subgroups of hospital mortality was assessed using the multivariable logistic regression model. All statistical analyses were conducted using R version 3.6.3 (2020, R Foundation for Statistical Computing, Vienna, Austria). A two-sided *p* value of 0.05 was considered significant.

## Results

### Study participants

**Fig. 1:**
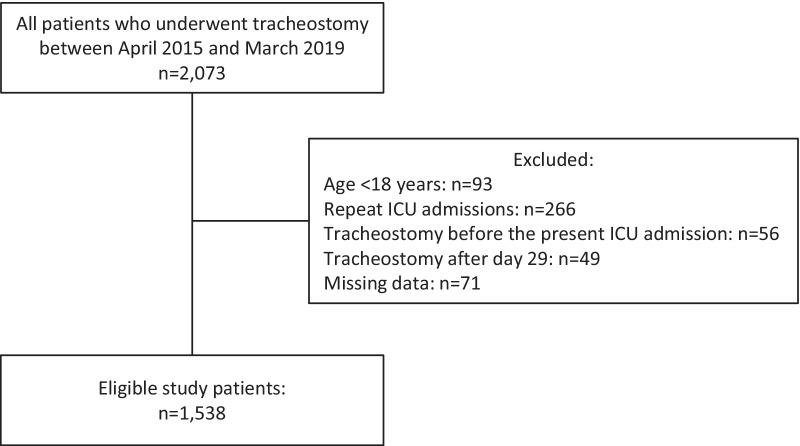
Patient inclusion flowchart. *ICU* intensive care unit

Among the 85558 admissions to 46 ICUs during the study period, 2073 patients who underwent tracheostomy and required mechanical ventilation were assessed for eligibility. Of them, we excluded 535 patients aged < 18 years (*n* = 93), with repeat ICU admissions (*n* = 266), with tracheostomy on ICU admission (*n* = 56), who underwent tracheostomy after day 29 (*n* = 49), and with missing data (*n* = 71). Finally, 1538 adult patients were included in the analysis (Fig. 1).

The characteristics of the study population divided by the quartiles of the timing of tracheostomy are presented in Table 1. The Q1 group was more likely to be admitted to the ICU for respiratory and neurological diagnoses than the other groups. In contrast, ICU admission for surgical, cardiac, and gastrointestinal diagnoses was more common for the Q4 group than for the other groups. Similar findings were observed in patients with chronic heart failure. In addition, the Q4 group had a lower PaO_2_:FiO_2_ and a higher GCS score within 24 h after ICU admission than those of the other groups. The frequency of comorbid maintenance dialysis, APACHE II score, and incidence of acute kidney injury within 24 h after ICU admission were the highest in the Q3 group. Patients who underwent tracheostomy were identified in 45 ICUs. The proportion of patients with the earliest tracheostomy classified as Q1 group ranged from 0 to 75% at each institution, and the timing of tracheostomy differed significantly between institutions (p < 0.001, Additional file 1).

**Table 1:** Patient characteristics by quartile of the timing of tracheostomy

	Q1 Tracheostomy ≤ 6 days (*n* = 446)	Q2 Tracheostomy 7–10 days (*n* = 405)	Q3 Tracheostomy 11–14 days (*n* = 323)	Q4 Tracheostomy > 14 days (*n* = 364)	*p* value
Age, years	70 (60–77)	70 (62–78)	70 (59–78)	70 (60–77)	0.658
Sex, male	308 (69.1%)	259 (64.0%)	207 (64.1%)	231 (63.5%)	0.277
Body mass index, kg/m^2^	21.6 (18.7–24.6)	21.8 (19.0–24.3)	22.0 (19.3–25.0)	22.1 (19.5–25.0)	0.176
Comorbidity					
Chronic heart failure	5 (1.1%)	5 (1.2%)	5 (1.5%)	14 (3.8%)	0.035
Chronic respiratory failure	26 (5.8%)	15 (3.7%)	11 (3.4%)	8 (2.2%)	0.065
Chronic liver disease	7 (1.6%)	6 (1.5%)	4 (1.2%)	5 (1.4%)	0.991
Malignancy	24 (5.4%)	23 (5.7%)	17 (5.3%)	22 (6.0%)	0.971
Immunodeficiency	40 (9.0%)	37 (9.1%)	43 (13.3%)	43 (11.8%)	0.158
Maintenance dialysis	12 (2.7%)	14 (3.5%)	25 (7.7%)	26 (7.1%)	0.001
Emergency admission	384 (86.1%)	360 (88.9%)	283 (87.6%)	299 (82.4%)	0.063
Surgical type of admission	172 (38.6%)	158 (39.0%)	124 (38.4%)	177 (48.6%)	0.010
Systematic diagnosis for ICU admission					
Cardiac	63 (14.1%)	80 (19.8%)	88 (27.2%)	162 (44.5%)	< 0.001
Respiratory	142 (31.8%)	109 (26.9%)	65 (20.1%)	77 (21.2%)	
Gastrointestinal	38 (8.5%)	51 (12.6%)	39 (12.1%)	49 (13.5%)	
Neurological	133 (29.8%)	103 (25.4%)	66 (20.4%)	33 (9.1%)	
Sepsis	8 (1.8%)	11 (2.7%)	13 (4.0%)	10 (2.7%)	
Trauma	37 (8.3%)	41 (10.1%)	27 (8.4%)	17 (4.7%)	
Other diagnosis	25 (5.6%)	10 (2.5%)	25 (7.7%)	16 (4.4%)	
APACHE II score	22 (17–28)	23 (19–29)	24 (19–31)	22 (18–28)	0.003
Data within 24 h after ICU admission					
Incidence of AKI	20/446 (4.5%)	23/404 (5.7%)	43/323 (13.3%)	43/363 (11.8%)	< 0.001
Lowest PaO_2_:FiO_2_	226 (154–320)	199 (133–293)	194 (125–291)	172 (117–236)	< 0.001
GCS score	12 (6–15)	11 (6–15)	13 (6–15)	15 (10–15)	< 0.001
Length of hospital stay before ICU admission, days	1 (0–6)	1 (0–4)	1 (0–6)	1 (0–5)	0.686

Data are presented as the median and interquartile range or as numbers (percentages)

*P* values are analyzed using the Chi-square (Fisher’s exact) test or the Kruskal–Wallis test

*ICU* intensive care unit, *APACHE* Acute Physiology and Chronic Health Evaluation, *AKI* acute kidney injury, *GCS* Glasgow Coma Scale

### Processes of care during ICU stay and length of hospital stay

The proportions of patients who received extracorporeal membrane oxygenation and continuous renal replacement therapy during ICU admission were higher in the Q4 group (18.7% and 44.2%, respectively) than in the other groups (*p* < 0.001 and *p* < 0.001, respectively; Table 2). In addition, the duration of mechanical ventilation in the ICU and the length of ICU stay were significantly longer in the Q4 group than in the other quartile groups (23.5 [IQR, 18.7–31.3] days and 27.5 [IQR, 21.7–35.0] days, respectively; *p* < 0.001 and *p* < 0.001, respectively). Moreover, the length of hospital stay was the longest in the Q4 group (78 [IQR, 51–119] days vs. 51 [IQR, 33–77] days for Q1; 56 [IQR, 36–90] days for Q2; and 64 [IQR, 44–103] days for Q3, *p* < 0.001).

**Table 2:** ICU treatment and patient outcomes by quartile of the timing of tracheostomy

	Q1 Tracheostomy ≤ 6 days	Q2 Tracheostomy 7–10 days	Q3 Tracheostomy 11–14 days	Q4 Tracheostomy > 14 days	*p* value
ECMO	18/446 (4.0%)	13/404 (3.2%)	24/323 (7.4%)	68/364 (18.7%)	< 0.001
CRRT	42/446 (9.4%)	62/404 (15.3%)	107/323 (33.1%)	161/364 (44.2%)	< 0.001
Duration of mechanical ventilation before tracheostomy, days	4 (3–5)	8 (7–9)	13 (12–14)	18 (16–21)	< 0.001
Duration of mechanical ventilation in ICU, days	5.6 (3.9–8.6)	10.8 (8.8–15.1)	15.2 (13.1–22.8)	23.5 (18.7–31.3)	< 0.001
Weaning from mechanical ventilation during ICU stay	297/444 (66.9%)	244/404 (60.4%)	197/323 (61.0%)	220/363 (60.6%)	0.149
Length of ICU stay, days	7.2 (5.0–11.0)	12.9 (10.1–17.2)	18.0 (14.6–26.7)	27.5 (21.7–35.0)	< 0.001
Length of hospital stay, days	51 (33–77)	56 (36–90)	64 (44–103)	78 (51–119)	< 0.001

Data are presented as the median and interquartile range or as numbers (percentages)

*P* values are analyzed using the Chi-square (Fisher’s exact) test or the Kruskal–Wallis test

*ECMO* extracorporeal membrane oxygenation, *CRRT* continuous renal replacement therapy, *ICU* intensive care unit

### Clinical outcomes

**Fig. 2:**
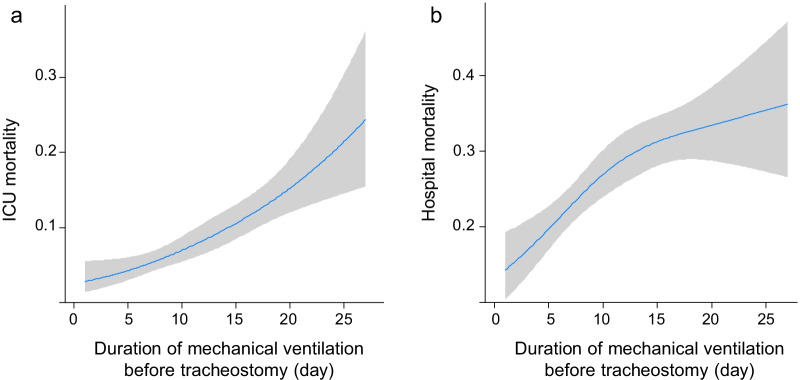
Mortality by the timing of tracheostomy. **a** ICU mortality; **b** hospital mortality. *ICU* intensive care unit

Hospital mortality, as the primary outcome, progressively increased with increasing quartiles of the timing of tracheostomy (Q1, 17.7%; Q2, 25.4%; Q3, 29.7%; Q4, 32.4%, *p* for trend < 0.001, Table 3). The adjusted multivariable analysis also showed a stepwise increase in hospital mortality rates across increasing quartiles (adjusted OR for quartile increment: 1.30, 95% CI: 1.17–1.44, *p* for trend < 0.001). The risk of hospital mortality was significantly higher in the Q4 group than in the Q1 group (adjusted OR: 2.26, 95% CI: 1.61–3.16). ICU mortality, as the secondary outcome, similarly showed a gradual increase as the quartile of the timing of tracheostomy increased (adjusted OR for quartile increment: 1.73, 95% CI: 1.45–2.07, *p* for trend < 0.001). The Q4 group also had a higher risk for ICU mortality than did the Q1 group (OR: 4.57, 95% CI: 2.59–8.04). Similarly, the restricted cubic spline showed a significant increase in ICU and hospital mortality with the timing of tracheostomy (Fig. 2). The overall hospital mortality rate at each institution varied from 0 to 42.9%, with significant differences between institutions (*p* = 0.027, Additional file 1).

**Table 3:** Patient outcomes by quartile of the timing of tracheostomy

	Q1 Tracheostomy ≤ 6 days	Q2 Tracheostomy 7–10 days	Q3 Tracheostomy 11–14 days	Q4 Tracheostomy > 14 days	OR for quartile increment (95% CI)	*p* value for trend
ICU mortality						
* n* (%)	17/444 (3.8%)	20/405 (4.9%)	29/322 (9.0%)	56/364 (15.4%)		
Crude OR (95% CI)	1 (reference)	1.30 (0.67–2.53)	2.49 (1.34–4.61)	4.57 (2.6–8.01)	1.72 (1.44–2.05)	< 0.001
Adjusted OR (95% CI) *	1 (reference)	1.25 (0.64–2.43)	2.34 (1.25–4.35)	4.57 (2.59–8.04)	1.73 (1.45–2.07)	< 0.001
Hospital mortality						
* n* (%)	79 (17.7%)	103 (25.4%)	96 (29.7%)	118 (32.4%)		
Crude OR (95% CI)	1 (reference)	1.58 (1.14–2.2)	1.96 (1.4–2.76)	2.23 (1.61–3.09)	1.29 (1.17–1.43)	< 0.001
Adjusted OR (95% CI)*	1 (reference)	1.52 (1.08–2.13)	1.82 (1.28–2.59)	2.26 (1.61–3.16)	1.30 (1.17–1.44)	< 0.001

*Adjusted OR for age, sex, APACHE II score in mortality

*OR* odds ratio, *CI* confidence interval, *ICU* intensive care unit

### Subgroup analyses

In total, 498 patients (32.4%) had a GCS score of < 8 on ICU admission (Table 4). Among them, hospital mortality tended to increase with increasing quartiles of the timing of tracheostomy (OR for quartile increment: 1.19, 95% CI: 0.98–1.44, *p* for trend = 0.081). In the 1040 patients with GCS score ≥ 8, there was a significant trend toward a stepwise increase in hospital mortality as the quartile of the timing of tracheostomy increased (OR for quartile increment: 1.33, 95% CI: 1.18–1.51, *p* for trend < 0.001). There was no significant interaction between the level of consciousness at ICU admission and hospital mortality (*p* = 0.310).

**Table 4:** Subgroup analysis for hospital mortality by quartile of the timing of tracheostomy

	Q1 Tracheostomy ≤ 6 days	Q2 Tracheostomy 7–10 days	Q3 Tracheostomy 11–14 days	Q4 Tracheostomy > 14 days	OR for quartile increment (95% CI)	*p* value for trend	*p* value for interaction*
Level of consciousness at ICU admission							0.310
GCS score < 8 (*n* = 498)							
* n* (%)	36/160 (22.5%)	32/161 (19.9%)	29/98 (29.6%)	24/79 (30.4%)			
Crude OR (95% CI)	1 (reference)	0.85 (0.5–1.46)	1.45 (0.82–2.56)	1.5 (0.82–2.76)	1.19 (0.98–1.44)	0.081	
GCS ≥ 8 (*n* = 1040)							
* n* (%)	43/286 (15.0%)	71/244 (29.1%)	67/225 (29.8%)	94/285 (33.0%)			
Crude OR (95% CI)	1 (reference)	2.32 (1.51–3.55)	2.4 (1.56–3.69)	2.78 (1.85–4.18)	1.33 (1.18–1.51)	< 0.001	
Diagnosis for ICU admission (respiratory failure)							0.218
Patients with respiratory failure (*n* = 834)							
*n* (%)	52/255 (20.4%)	74/238 (31.1%)	62/172 (36.0%)	56/169 (33.1%)			
Crude OR (95% CI)	1 (reference)	1.76 (1.17–2.65)	2.2 (1.42–3.4)	1.93 (1.24–3.01)	1.25 (1.09–1.43)	0.001	
Patients without respiratory failure (*n* = 704)							
* n* (%)	27/191 (14.1%)	29/167 (17.4%)	34/151 (22.5%)	62/195 (31.8%)			
Crude OR (95% CI)	1 (reference)	1.28 (0.72–2.26)	1.77 (1.01–3.08)	2.83 (1.71–4.7)	1.43 (1.21–1.67)	< 0.001	
Diagnosis for ICU admission (cardiovascular surgery)							0.681
Patients after cardiovascular surgery (*n* = 202)							
* n* (%)	2/13 (15.4%)	5/32 (15.6%)	13/45 (28.9%)	35/112 (31.3%)			
Crude OR (95% CI)	1 (reference)	1.02 (0.17–6.11)	2.23 (0.43–11.5)	2.5 (0.53–11.9)	1.41 (0.98–2.03)	0.063	
Other patients (*n* = 1336)							
* n* (%)	77/433 (17.8%)	98/373 (26.3%)	83/278 (29.9%)	83/252 (32.9%)			
Crude OR (95% CI)	1 (reference)	1.65 (1.18–2.31)	1.97 (1.38–2.81)	2.27 (1.58–3.26)	1.3 (1.17–1.46)	< 0.001	

*p value for interaction is calculated between the timing of the tracheostomy and the diagnosis for ICU admission

*OR* odds ratio, *CI* confidence interval, *ICU* intensive care unit, *GCS* Glasgow Coma Scale

Meanwhile, 834 patients (54.2%) presented with respiratory failure on ICU admission. In the same group of patients, hospital mortality was significantly higher in the Q4 group than in the Q1 group (crude OR: 1.93, 95% CI: 1.24–3.01). Hospital mortality increased progressively with the increase in the quartile of the timing of tracheostomy in both patients with and without respiratory failure (*p* for trend = 0.001 and < 0.001, respectively; *p* for interactio*n* = 0.218).

Furthermore, 202 patients (13.1%) were admitted to the ICUs after cardiovascular surgery. Hospital mortality tended to increase as the quartile of the timing of tracheostomy increased among these patients (OR for quartile increment: 1.41, 95% CI: 0.98–2.03, *p* for trend = 0.063). A similar trend toward a stepwise increase in hospital mortality rates across increasing quartiles was shown among those not admitted to the ICUs after cardiovascular surgery (OR for quartile increment: 1.3, 95% CI: 1.17–1.46, *p* for trend < 0.001; *p* for interactio*n* = 0.681).

## Discussion

### Key findings

This multi-institutional observational study evaluated the clinical impact of the timing of tracheostomy on patient outcomes. The results showed that patients with delayed tracheostomy had prolonged mechanical ventilation and longer ICU and hospital stays. Extended mechanical ventilation prior to tracheostomy was associated with a high mortality rate in a time-dependent manner. This trend was the same for different diagnoses and levels of consciousness at the time of ICU admission.

### Relationship with previous studies

Tracheostomy is a widely used alternative to translaryngeal endotracheal intubation in critical care settings. It can be performed both at the bedside and in the operating room; thus, its implementation is highly feasible [25].

The consensus established in 1989 recommends that mechanical ventilation by translaryngeal endotracheal intubation be performed in patients expected to be extubated within 10 days, while those expected to be mechanically ventilated for more than 21 days should be placed on tracheostomy [9]. Accordingly, tracheostomy is generally considered within 2 weeks after the commencement of mechanical ventilation in critically ill patients [10, 26, 27]. In the current study, 76.3% (1174 of 1538) of the patients were tracheostomized within the first 2 weeks. However, the international expert task force in 2017 has yet to specify the optimal timing of tracheostomy [28]. Furthermore, recent global guidelines on acute respiratory failure also do not address tracheostomy itself despite its integral role in the management of mechanical ventilation [29, 30].

Tracheostomy has been associated with patient outcomes, such as lengths of ICU and hospital stay, duration of mechanical ventilation, and cost, but large randomized controlled trials have not established an association with mortality [20, 31–33]. The largest randomized controlled trial, the TracMan trial conducted in the United Kingdom, compared early (≤ 4 days) and late tracheostomy (≥ 10 days) in 909 patients expected to require mechanical ventilation for at least 7 days [31]. Their results showed similar 30-day mortality between the early and late tracheostomy groups (30.8% vs. 31.5%). The mortality rates for other periods up to 2 years were also similar, substantially influencing the meta-analysis that included the study. Of the 454 patients in the early tracheostomy group in the TracMan trial, 385 (84.6%) underwent tracheostomy within 4 days of admission to the critical care unit. However, of the 454 patients in the late tracheostomy group, 244 (53.7%) did not undergo tracheostomy due to ICU discharge or weaning from mechanical ventilation. Meanwhile, 33 patients (7.3%) underwent tracheostomy before day 10, contrary to the protocol. Thus, the difficulty in predicting the required duration of mechanical ventilation has added an element of impracticality to the conduct of appropriate interventional studies [28].

Several large observational studies have described significant differences in patient outcomes, depending on the timing of tracheostomy. Patel et al. conducted a secondary analysis of a historical cohort of 1175 tracheostomy patients in the United States and found that mortality was 1.4-fold higher in late tracheostomy (≥ 14 days) than in early tracheostomy (< 14 days) [34]. Furthermore, a large multicenter study of 43,916 ICU patients reported that the timing of tracheostomy was significantly correlated with the duration of mechanical ventilation and length of ICU and hospital stay [35]. Our present study is one of the largest observational studies, and our investigation of a graded classification of the timing of tracheostomy showed a stepwise relationship between the timing of tracheostomy and various patient outcomes, including mortality.

Consistent with previous studies, the present study found that early tracheostomy was beneficial in the subgroups of patients requiring mechanical ventilation. Pinheiro et al. conducted a single-center cohort study of Brazilian patients with acute severe brain injury presenting with a GCS score < 8 and found that 28-day mortality was significantly lower in the early (≤ 8 days) tracheostomy group than in the late (> 8 days) tracheostomy group (9% vs. 47%) [36]. In subsequent studies of brain injury, early tracheostomy was found to shorten the duration of treatment (mechanical ventilation and lengths of ICU and hospital stay) and improve neurological outcomes, although the association with mortality remains potentially insignificant [37, 38].

Regarding the relationship between tracheostomy and patient outcomes in acute respiratory failure, a secondary analysis of the LUNG-SAFE study [39], an international cohort study of acute respiratory distress syndrome, was conducted [12]. Although the 60- and 90-day mortality rates were similar, the treatment duration (duration of mechanical ventilation, and lengths of ICU and hospital stay) and 28-day mortality rate (22.0% vs. 10.9%) were significantly lower in the early tracheostomy group (< 7 days) than in the late tracheostomy group (≥ 8 days). Other studies on patients with venovenous extracorporeal membrane oxygenation [40] or coronavirus disease 2019 [41] similarly found that early tracheostomy has a favorable impact on patient outcomes, mainly of shortening the duration of treatment. Our findings showed that early tracheostomy was associated with favorable patient outcomes in these subgroups as well as in the existing literature, and our study with a large population demonstrated that it also had a positive impact on mortality.

The other subgroup to consider is postoperative patients after cardiovascular surgery, who may require prolonged mechanical ventilation due to older age or more comorbidities [42]. However, concerns about surgical site infection and mediastinitis have led to the consideration of tracheostomy for patients who required mechanical ventilation for more than 2 weeks [43] and their optimal timing of tracheostomy is under investigation. Recently, Okada et al. reported an observational study of patients after cardiovascular surgery through a median sternotomy at a university hospital in Japan [44]. Compared with the late tracheostomy group (> 7 days after cardiovascular surgery), the early tracheostomy group (≤ 7 days) showed a shorter duration of ventilatory management and a higher 1-year survival rate (65% vs. 31%) without increased morbidities. This multicenter study using a nationwide database also demonstrated a stepwise association between the timing of tracheostomy and mortality.

Thus, the timing of tracheostomy was similarly associated with hospital mortality in all subgroups examined in this study. However, the main causes of death and detailed complications during mechanical ventilation were not obtained in our data, and the actual contribution of late tracheostomy to increased mortality and prolonged duration of treatment was not determined. Delayed indication of tracheostomy and prolonged translaryngeal endotracheal intubation may require prolonged administration of sedatives and opioids [45]. Therefore, late tracheostomy may lead to slower weaning from mechanical ventilation and a higher incidence of VAP, a critical complication of mechanical ventilation associated with morbidity and mortality [21, 46]. Indeed, a recent systematic review of randomized control studies which compare early (≤ 7 days) vs. late (> 7 days) tracheotomy demonstrated that late tracheostomy resulted in higher VAP incidence and longer duration of mechanical ventilation and ICU stay [17]. These factors may contribute to the increased mortality of late tracheostomy; however, the timing of tracheostomy and hospital mortality varied among the registered institutions, suggesting that differences in treatment strategies and facilities may have an impact on mortality. Further investigation, including detailed treatment and complications during mechanical ventilation in individual patients, is warranted.

### Implications of study findings

Using data from the JIPAD, our findings indicate that the timing of tracheostomy is significantly associated with mortality. Compared with late tracheostomy, early tracheostomy after the initiation of mechanical ventilation is associated with a significantly high probability of favorable patient prognosis. Tracheostomy is a common and widely accepted procedure, and early tracheostomy can be used to improve patient prognosis. Furthermore, our findings provide the rationale for further investigation into the optimal timing of tracheostomy.

### Strengths and limitations

The strength of this study is that it is a large multicenter cohort study that is inclusive and representative of numerous institutions and thus allows generalizability. However, our study also had some limitations. First, considering the nature of the observational study design, the results of this analysis are prone to misinterpretation and confounding that cannot be measured. Second, the indications and procedures for tracheostomy were based on institutional protocol, with no standardization. Third, the database used in this study contained treatment records during the stay at each registered institution. Therefore, our data did not provide information on treatment, including mechanical ventilation, prior to ICU admission and after the discharge. Fourth, the registry did not include information on the respiratory status or concomitant therapy at the time of tracheostomy. However, detailed clinical information on ICU admission enabled us to adjust our analysis for several major confounders using logistic regression models, and subgroup analyses showed robust associations with clinical outcomes.

## Conclusion

Our study showed that the timing of tracheostomy is significantly associated with mortality in a stepwise manner. A similar trend was observed across all levels of consciousness and diagnoses at ICU admission. These findings support the need for early tracheostomy in critically ill patients requiring mechanical ventilation, with an emphasis on patient outcomes.

## Supplementary Information

**Additional file 1. **Patients who underwent tracheostomy and overall hospital mortality at each institution.

## Data Availability

The data that support the findings of this study are available from the JIPAD project, but restrictions apply to the availability of these data, which were used under license for the current study, and so are not publicly available.

## Abbreviations

ANZICS APD: Australian and New Zealand Intensive Care Society Adult Patient Database
APACHE: Acute Physiology and Chronic Health Evaluation
CI: Confidence interval
GCS: Glasgow Coma Scale
ICU: Intensive care unit
IQR: Interquartile ranges
JIPAD: Japanese Intensive Care PAtient Database
OR: Odds ratio
VAP: Ventilator-associated pneumonia
