# Supplementary material for: Association between early tracheostomy and patient outcomes in critically ill patients on mechanical ventilation: a multicenter cohort study
Source: J Intensive Care. 2022 Apr 11;10:19. doi: 10.1186/s40560-022-00610-x (PMC8996211; doi:10.1186/s40560-022-00610-x)
Supplement: Supplementary file 1 — Additional file 1. Patients who underwent tracheostomy and overall hospital mortality at each institution. [file 40560_2022_610_MOESM1_ESM.docx]

**Additional file 1.** Patients who underwent tracheostomy and overall hospital mortality at each institution

|  | Q1  Tracheostomy ≤6 days | Q2 Tracheostomy 7–10 days | Q3  Tracheostomy 11–14 days | Q4  Tracheostomy >14 days | P value | Hospital  mortality | P value |
| --- | --- | --- | --- | --- | --- | --- | --- |
| Site 1 | 15/41 (36.6%) | 13/41 (31.7%) | 6/41 (14.6%) | 7/41 (17.1%) | <0.001 | 6/41 (14.6%) | 0.027 |
| Site 2 | 4/24 (16.7%) | 3/24 (12.5%) | 9/24 (37.5%) | 8/24 (33.3%) |  | 5/24 (20.8%) |  |
| Site 3 | 15/59 (25.4%) | 19/59 (32.2%) | 8/59 (13.6%) | 17/59 (28.8%) |  | 14/59 (23.7%) |  |
| Site 4 | 16/52 (30.8%) | 13/52 (25.0%) | 13/52 (25.0%) | 10/52 (19.2%) |  | 16/52 (30.8%) |  |
| Site 5 | 11/40 (27.5%) | 11/40 (27.5%) | 7/40 (17.5%) | 11/40 (27.5%) |  | 11/40 (27.5%) |  |
| Site 6 | 27/65 (41.5%) | 15/65 (23.1%) | 12/65 (18.5%) | 11/65 (16.9%) |  | 13/65 (20.0%) |  |
| Site 7 | 13/51 (25.5%) | 8/51 (15.7%) | 10/51 (19.6%) | 20/51 (39.2%) |  | 14/51 (27.5%) |  |
| Site 8 | 18/60 (30.0%) | 14/60 (23.3%) | 14/60 (23.3%) | 14/60 (23.3%) |  | 11/60 (18.3%) |  |
| Site 9 | 12/47 (25.5%) | 9/47 (19.1%) | 9/47 (19.1%) | 17/47 (36.2%) |  | 8/47 (17.0%) |  |
| Site 10 | 21/60 (35.0%) | 19/60 (31.7%) | 10/60 (16.7%) | 10/60 (16.7%) |  | 17/60 (28.3%) |  |
| Site 11 | 8/39 (20.5%) | 12/39 (30.8%) | 14/39 (35.9%) | 5/39 (12.8%) |  | 4/39 (10.3%) |  |
| Site 12 | 19/63 (30.2%) | 17/63 (27.0%) | 12/63 (19.0%) | 15/63 (23.8%) |  | 22/63 (34.9%) |  |
| Site 13 | 19/93 (20.4%) | 23/93 (24.7%) | 21/93 (22.6%) | 30/93 (32.3%) |  | 26/93 (28.0%) |  |
| Site 14 | 9/22 (40.9%) | 8/22 (36.4%) | 1/22 (4.5%) | 4/22 (18.2%) |  | 3/22 (13.6%) |  |
| Site 15 | 2/17 (11.8%) | 4/17 (23.5%) | 5/17 (29.4%) | 6/17 (35.3%) |  | 4/17 (23.5%) |  |
| Site 16 | 21/85 (24.7%) | 23/85 (27.1%) | 18/85 (21.2%) | 23/85 (27.1%) |  | 22/85 (25.9%) |  |
| Site 17 | 12/50 (24.0%) | 21/50 (42.0%) | 9/50 (18.0%) | 8/50 (16.0%) |  | 16/50 (32.0%) |  |
| Site 18 | 7/27 (25.9%) | 8/27 (29.6%) | 8/27 (29.6%) | 4/27 (14.8%) |  | 8/27 (29.6%) |  |
| Site 19 | 5/38 (13.2%) | 9/38 (23.7%) | 10/38 (26.3%) | 14/38 (36.8%) |  | 14/38 (36.8%) |  |
| Site 20 | 16/34 (47.1%) | 9/34 (26.5%) | 3/34 (8.8%) | 6/34 (17.6%) |  | 13/34 (38.2%) |  |
| Site 21 | 1/4 (25.0%) | 1/4 (25.0%) | 1/4 (25.0%) | 1/4 (25.0%) |  | 1/4 (25.0%) |  |
| Site 22 | 8/29 (27.6%) | 6/29 (20.7%) | 5/29 (17.2%) | 10/29 (34.5%) |  | 12/29 (41.4%) |  |
| Site 23 | 5/13 (38.5%) | 5/13 (38.5%) | 2/13 (15.4%) | 1/13 (7.7%) |  | 3/13 (23.1%) |  |
| Site 24 | 4/16 (25.0%) | 3/16 (18.8%) | 7/16 (43.8%) | 2/16 (12.5%) |  | 2/16 (12.5%) |  |
| Site 25 | 9/16 (56.3%) | 4/16 (25.0%) | 3/16 (18.8%) | 0/16 (0%) |  | 5/16 (31.3%) |  |
| Site 26 | 4/12 (33.3%) | 4/12 (33.3%) | 1/12 (8.3%) | 3/12 (25.0%) |  | 0/12 (0%) |  |
| Site 27 | 34/55 (61.8%) | 12/55 (21.8%) | 4/55 (7.3%) | 5/55 (9.1%) |  | 14/55 (25.5%) |  |
| Site 28 | 32/78 (41.0%) | 16/78 (20.5%) | 17/78 (21.8%) | 13/78 (16.7%) |  | 24/78 (30.8%) |  |
| Site 29 | 8/53 (15.1%) | 12/53 (22.6%) | 15/53 (28.3%) | 18/53 (34.0%) |  | 19/53 (35.8%) |  |
| Site 30 | 1/11 (9.1%) | 6/11 (54.5%) | 1/11 (9.1%) | 3/11 (27.3%) |  | 0/11 (0%) |  |
| Site 31 | 9/59 (15.3%) | 22/59 (37.3%) | 18/59 (30.5%) | 10/59 (16.9%) |  | 7/59 (11.9%) |  |
| Site 32 | 9/31 (29.0%) | 8/31 (25.8%) | 6/31 (19.4%) | 8/31 (25.8%) |  | 11/31 (35.5%) |  |
| Site 33 | 3/11 (27.3%) | 3/11 (27.3%) | 3/11 (27.3%) | 2/11 (18.2%) |  | 0/11 (0%) |  |
| Site 34 | 1/5 (20.0%) | 1/5 (20.0%) | 0/5 (0%) | 3/5 (60.0%) |  | 2/5 (40.0%) |  |
| Site 35 | 10/18 (55.6%) | 5/18 (27.8%) | 1/18 (5.6%) | 2/18 (11.1%) |  | 5/18 (27.8%) |  |
| Site 36 | 2/18 (11.1%) | 2/18 (11.1%) | 7/18 (38.9%) | 7/18 (38.9%) |  | 5/18 (27.8%) |  |
| Site 37 | 4/20 (20.0%) | 4/20 (20.0%) | 6/20 (30.0%) | 6/20 (30.0%) |  | 7/20 (35.0%) |  |
| Site 38 | 2/4 (50.0%) | 1/4 (25.0%) | 1/4 (25.0%) | 0/4 (0%) |  | 1/4 (25.0%) |  |
| Site 39 | 2/25 (8.0%) | 7/25 (28.0%) | 8/25 (32.0%) | 8/25 (32.0%) |  | 6/25 (24.0%) |  |
| Site 40 | 12/41 (29.3%) | 16/41 (39.0%) | 8/41 (19.5%) | 5/41 (12.2%) |  | 9/41 (22.0%) |  |
| Site 41 | 2/10 (20.0%) | 1/10 (10.0%) | 1/10 (10.0%) | 6/10 (60.0%) |  | 2/10 (20.0%) |  |
| Site 42 | 4/14 (28.6%) | 3/14 (21.4%) | 3/14 (21.4%) | 4/14 (28.6%) |  | 6/14 (42.9%) |  |
| Site 43 | 0/12 (0%) | 3/12 (25.0%) | 3/12 (25.0%) | 6/12 (50.0%) |  | 4/12 (33.3%) |  |
| Site 44 | 7/12 (58.3%) | 2/12 (16.7%) | 2/12 (16.7%) | 1/12 (8.3%) |  | 4/12 (33.3%) |  |
| Site 45 | 3/4 (75.0%) | 0/4 (0%) | 1/4 (25.0%) | 0/4 (0%) |  | 0/4 (0%) |  |
| Data are presented as numbers (percentages).  P values are analyzed using Fisher’s exact test for the quartile of the timing of tracheostomy and mortality rate. | | | | | | | |
